# Online Black-Markets: An Investigation of a Digital Infrastructure in the Dark

**DOI:** 10.1007/s10796-021-10187-9

**Published:** 2021-09-21

**Authors:** Paolo Spagnoletti, Federica Ceci, Bendik Bygstad

**Affiliations:** 1 Department of Business and Management, Luiss University, Rome, Italy; 2 Department of Economics and Management, G. d’Annunzio University, Pescara, Italy; 3 Department of Informatics, University of Oslo, Oslo, Norway

**Keywords:** Darknet, Digital infrastructure, Social commerce, Resilience, Marketplace

## Abstract

This paper investigates the functioning of Online Black-Markets (OBMs), i.e. a digital infrastructure operating in the Dark Net that enables the exchange of illegal goods such as drugs, weapons and fake digital identities. OBMs exist notwithstanding adverse conditions such as police interventions, scams and market breakdowns. Relying on a longitudinal case study, we focus on the dynamics of interactions among actors and marketplace technologies and we identify three mechanisms explaining OBMs operations. In particular, we show that OBMs infrastructure is the result of commoditization, platformization and resilience processes. Our contribution relies on the identification of community-based mechanisms that generate the OBMs infrastructure, extending the current understanding of e-commerce and social commerce.

## Introduction

In October 2013 the FBI closed down *Silk Road*, the first online illegal marketplace, which served around 100.000 customers, primarily buying illegal drugs. In a much-publicised trial, the founder received a life sentence, and the US Government has later seized more than 1 bn worth of bitcoin (The Guardian, Nov 6th, 2020). However, *Silk Road* seizure and the arrest of its admin did not stop buyers and vendors: clone marketplaces appeared few weeks later and several new, more technically robust, marketplaces opened in the Dark Net in the subsequent months (Aldridge & Décary-Hétu, 2016). In the period from October 2013 until April 2018, we registered the existence of 122 illegal marketplaces. In January 2021 German police closed down the *DarkMarket* marketplace*,* with 2.400 sellers of drugs, stolen credit card data, and malware*,* which was reported to serve half a million users (The Guardian Jan 12th 2021). How are these on-going illegal activities possible, in a world characterized by Internet surveillance (Samtani, Chinn, Chen, & Nunamaker, 2017; Zuboff, 2015)?

The answer relies in the existence of the Dark Net, that are layers of the Internet that guarantee the anonymity of online interactions (Chaudhry, 2017; Li & Whinston, 2020). The Dark Net can be accessed only with specific software such as the Tor browser; web pages in the Dark Net are not indexed by search engines and access to hidden services cannot be traced (Chertoff, 2017). Within the Dark Net, an infrastructure of tools and services for electronic commerce emerged: the Online Black-Markets (OBMs). OBMs consist of computing and network resources (i.e. cryptocurrencies, the Tor network) that connect buyers and vendors interested in exchanging specific goods, mainly illegal products and services. Coordination and transactions in OBMs are supported by Online Marketplaces (OMs) similar to those enabling legitimate e-commerce and social commerce. Today, the OBMs infrastructure is a global scale phenomenon: one study reported a volume of $220 million in transactions on a single marketplace (Soska & Christin, 2015), while more recent studies estimate over $790 million (Chainanalysis, 2020). OBMs operate in absence of formal rules, legal protection, social legitimacy and despite conflicting goals among actors. In fact, a variety of actors interacts in OBMs: marketplace users and admins, hackers, software developers, and law enforcement agents (Beckert & Wehinger, 2013; Décary-Hétu & Giommoni, 2017; Lacson & Jones, 2016; Paquet-Clouston, Décary-Hétu, & Morselli, 2018; Van Buskirk et al., 2017).

From a research point of view, OBMs represent a unique setting due to unobservability of its technological and human components, heterogeneous and adverse forces influencing infrastructural growth, presence of unknown actors with conflicting goals, and a negative global impact of its social outcomes. We investigate the phenomenon of OBMs using the theoretical lens of digital infrastructure. In line with the definition provided by Constantinides, Henfridsson and Parker (2018), a digital infrastructure is composed by “*computing and network resources that allow multiple stakeholders to orchestrate their service and content needs*” (Constantinides et al., 2018). The OBMs digital infrastructure generates websites, marketplaces, online forums, security technologies and other means supporting complex sociotechnical interactions for illegal purposes (Huang, Siegel, & Madnick, 2018; Li & Whinston, 2020). The dynamics of such tightly coupled interactions are generated by underlying mechanisms that are not always observable and therefore partially unknown.

In this study we document the functioning of OBMs, and we investigate the underlying forces and mechanisms explaining the existence of the OBMs infrastructure despite law enforcement efforts. In doing so, we focus on the generative dynamics of tightly coupled interactions among actors and their supporting computing and network resources. The misalignment of actors’ goals may challenge the development and adoption of the most appropriate anonymity tools and their enabling technologies. When goals are in conflict, the self-interest of actors may represent a threat for infrastructure operations. In line with this reasoning, we aim to answer the following research question: *what are the generative mechanisms of the Online Black-Markets infrastructure?*

From a methodological point of view, we conduct a longitudinal case study focusing on the evolution of technologies and social practices. Our research approach is a critical realist case study (Wynn & Williams, 2012), chosen for two reasons. First, the OBMs phenomenon calls for an interdisciplinary and multi-level conceptualization of the OBMs, requiring a multi-method research design, which is well supported within critical realism (Dobson, 2001; Mingers, 2001). Second, with the present work we aim to move our understanding far beyond a scattered and anecdotical view of OBMs and aim at revealing the underlying mechanisms that shape this special form of digital infrastructure over time (Wynn & Williams, 2012). We build our dataset by triangulating archival data from secondary sources (e.g. public reports; scientific papers; websites; press documents) with primary data obtained from interviews with Law Enforcement Agency (LEA) agents, and analyses of illegal offerings. Our analysis reveals three causal mechanisms operating in the OBMs infrastructure: commoditization, platformization and resilience. Our contribution is that the OBMs infrastructure is generated by three interacting mechanisms; they show that the technical and social elements not only interact to facilitate transactions, rather they *constitute* OBMs, feeding on each other. Moreover, we shed light on the infrastructural mechanisms supporting OBMs operations and discuss the implications for e-commerce and social commerce.

The paper is structured as follows. First, we review the literature on OMs and OBMs. Next, we frame the OBMs as a digital infrastructure. The research method is illustrated in section 4. Our empirical findings are then presented to introduce the mechanisms explaining the OBMs operations. A discussion section closes the paper by drawing conclusions about both theoretical and practical contributions.

## Background Literature

We draw on existing IS research to introduce the main features of OMs and review the primary factors influencing the performances of OMs. We then focus on social, technological and value-creating mechanisms explaining the functioning of OMs. Finally, we review the literature on OBMs to compare their mechanisms with OMs and evaluate how OBMs operate despite the adverse institutional conditions.

### Online Marketplaces

Online marketplaces are intermediation structures that facilitate transactions through online media. OMs provide functions to aggregate and match suppliers and customers, enhance trust, and share market information (O’Reilly & Finnegan, 2010). OMs also provide value-added communication, brokerage and integration services for buyers and sellers by supporting basic market functions, meeting management needs for information and process support, and operating the IT infrastructure (ibid, p. 463). The increased popularity of social media has extended the scope of OMs and added new collaborative and user-centered functions, leading to the rise of social commerce (Huang & Benyoucef, 2013). In social commerce, the delivery of e-commerce activities and transactions is influenced by community interactions such as, for instance, the rating and recommendations issued by users on goods and vendors (Liang & Turban, 2011). Therefore, we can conclude that OMs are socio-technical structures that facilitate coordination and transactions among buyers and sellers through online media.

To succeed in the market, OMs must continuously update and improve their structures and leverage strategic, institutional and social factors. Marketplace administrators must choose appropriate governance structures, service provision strategies, organizational capabilities and strategic manipulation of OM operations (Wang, Zheng, Xu, Li, & Meng, 2008). For instance, product offerings can be expanded with innovative products to positively impact volumes traded and enhance revenues. Moreover, service provision strategies must be adapted to product characteristics such as in the case of auctions and electronic catalogues that fit with commodities whereas supply chain services are more suitable for bespoke products (Hopkins & Kehoe, 2006). Another example is technology arrangements like shared databases and IT systems for routing orders between trading partners that improve OM performances through timely information sharing and reduced transaction processing costs (Wang & Archer, 2004).

Institutional and social factors also influence the performance of OMs. Trust plays an important role among these factors, since it enables shared expectations between unknown social actors who have no experience of previous interactions (McKnight, Choudhury, & Kacmar, 2002). Therefore, OMs must ensure that transactions are securely completed and that both parties to the transaction, buyers and sellers, do not partake in opportunistic behaviors (Pavlou & Gefen, 2004). Online buyer behavior is shaped by the beliefs that a marketplace will institute and enforce rules and procedures to mitigate the risk of opportunistic behaviors (Pavlou & Gefen, 2004). Various forms of institutional mechanisms have been implemented by OMs, including escrow services, credit card guarantees and privacy protections that often require the involvement of authorities and third parties to generate proper transactional conditions (Lu, Zeng, & Fan, 2016). When such mechanisms are in place, buyers can trust the marketplace administrator, the community of sellers and the local e-commerce environment (Kim & Ahn, 2007; Lu et al., 2016; Pavlou & Gefen, 2004).

The success of OMs is also contingent on the level of participation and use of OMs’ functions. In addition to institution-based trust, online purchase intentions are influenced by the perceived social presence of a marketplace and the perceived social presence of others (Lu et al., 2016). For instance, when unanticipated market shocks reduce the number and types of traders utilizing OMs the lack of participation hinders OM performances (O’Reilly & Finnegan, 2010; Wang et al., 2008).

OMs leverage the value-creating mechanisms of other digital platforms (de Reuver, Sørensen, & Basole, 2017; Ghazawneh & Henfridsson, 2015; Hein et al., 2020; Spagnoletti, Resca, & Lee, 2015). These value-creating mechanisms build on the efficient facilitation of transactions (Tiwana, 2014) and the provision of features enabling innovation (Yoo, Henfridsson, & Lyytinen, 2010). OMs act as intermediaries by directly matching supply to demand and suggesting possible transactions or by providing easy-to-use search functions through which users can find transaction partners. Via the orchestration of transactions, digital platforms create two-sided markets (Armstrong, 2006) that leverage cross-side network effects. The basis for this value-creating mechanism is a modular software-based platform, where the platform owner provides value-creating services, such as payment functionalities or recommender systems to increase the efficiency and convenience of the services for the ecosystem (Hein et al., 2020).

### Online Black-Markets

Online Black-Markets (OBMs), also referred to as darknet marketplaces or cryptomarkets in the literature (Aldridge & Décary-Hétu, 2016; Bhaskar, Linacre, & Machin, 2017; Chaudhry, 2017), are a sociotechnical structure of systems, people and organizations. OBMs are composed by anonymous marketplaces that connect buyers and vendors interested in the exchange of illegal products and services through the use of technologies (i.e. cryptocurrencies, the Tor network and other anonymizing services), developed by communities that design, implement, maintain and adopt its functionalities. The OBMs operations are contrasted by severe adverse conditions, such as absence of formal rules, law enforcement control, legal protection, social legitimacy (Beckert & Wehinger, 2013; Décary-Hétu & Giommoni, 2017; Lacson & Jones, 2016; Paquet-Clouston et al., 2018; Van Buskirk et al., 2017). Moreover, OBMs are characterized by conflicting goals among actors that undermine their functioning, namely police operations, attack of hackers and opportunistic behaviour of marketplace administrators or vendors (Bhaskar et al., 2017; Soska & Christin, 2015). However, such shocks do not affect the existence of OBMs (Décary-Hétu & Giommoni, 2017).

The research on OBMs related phenomena is limited, but relevant for our study. One reason for the scarcity of research is the challenge of accessing the empirical field (Victor Benjamin, Valacich, & Chen, 2019). Another is that the conceptualization of the domain has not yet stabilized, and current studies either focus on online illegal behaviors or on tools supporting fraudulent interactions rather than developing a holistic understanding of the OBM as a complex socio-technical phenomenon.

Previous research can be grouped into three streams as illustrated in Table 1. A first stream of literature is focused on the interactions of actors performing illegal activities on OBMs. Drugs, malware and stolen data are examples of goods exchanged in forums and online marketplaces (Odabas, Holt, & Breiger, 2017; Samtani et al., 2017; Soska & Christin, 2015). These studies issue recommendations for policymakers in specific domains such as cyber-intelligence and cyber-defense. Despite their practitioner orientation, most of these works draw on rigorous empirical analysis based on methods and techniques to analyze data collected through web-crawling (i.e. the use of software agents that systematically browse the internet to download and index specific content) and nonobtrusive netnographic observations (Bhaskar et al., 2017; Christin, 2012). For the purposes of this study, we refer to this stream as a valuable source of secondary data.

**Table 1:** Summary of OMs and OBMs studies

Research focus	Key concepts in OMs studies	Key concepts in OBMs studies
Interactions	Transaction behavior, advertising, referring and recommending, social influence, co-creation (Huang and Benyoucef, 2013; Liang and Turban, 2011)	Variations in sales, vendors, product categories, ratings (Odabas et al. 2017; Samtani et al. 2017; Soska and Christin 2015)
Technology	Escrow, credit card guarantees, collaborative supply chain, social networking sites (Huang and Benyoucef, 2013; Pavlou and Gefen, 2004; Wang and Archer, 2004)	Escrow, cryptocurrencies, Tor network, Public Key Infrastructures, Internet Relay Chat, forums (Benjamin et al. 2016; Van Hout and Bingham 2014; Leukfeldt et al. 2017)
Value-creation	Transaction platform, innovation platform, cross-side network effects (Ghazawneh and Henfridsson, 2015; Hein et al., 2020)	Value chain, cash-out, management and innovation dynamics (Bakken et al. 2017; Huang et al. 2018; Kraemer-Mbula et al. 2013; van Wegberg et al. 2018)

A second stream of research focuses on technologies and tools used for communication and trade among anonymous members of the OBMs communities. In particular, online meeting places such as forums and Internet Relay Chat (IRC) are analyzed to show how these artifacts support user participation (Benjamin, Zhang, Nunamaker, & Chen, 2016; Leukfeldt, Kleemans, & Stol, 2017). As for the trade, OBMs host many marketplaces, whose functions include product listings, ratings, wallets and escrow services. The security of transactions is guaranteed by public key infrastructures and decentralized systems such as cryptocurrencies (Me, Spagnoletti, & Pesticcio, 2017). Moreover, such marketplaces have replaced many online forums supporting anonymous interactions between buyers and sellers of illegal goods. The aim of these studies is to explore the ways in which marketplaces operate (Van Hout & Bingham, 2014). Criminal case studies, netnographic observations and, in some cases interviews with buyers and vendors, have been conducted to explain how marketplace functions enable users to interact online.

A third stream analyses value-creation in OBMs. These studies use managerial concepts such as value chains and transaction costs to investigate business models in criminal networks (Bakken, Moeller, & Sandberg, 2017; K. Huang et al., 2018; Kraemer-Mbula, Tang, & Rush, 2013; van Wegberg et al., 2018). The main contribution of these studies is to shed light on commonalities and differences between online and conventional black markets and to explain recent trends such as the commoditization of criminal services. Some studies investigate the relationship between OBMs technologies and their social organization. Their focus has been mostly concentrated on functioning and operations over limited time spans.

OBMs experience multiple and frequent disruptions. Technical failure of hidden services may be either injected by police agents engaged in law enforcement operations or may be caused by hackers and internal scammers. Notwithstanding these failures, the OBMs infrastructure has shown exceptional capabilities to react and persist over time. Only a few studies have investigated the effects of disruptions and adverse forces on OBMs mostly focusing on their criminological implications (Décary-Hétu & Giommoni, 2017; Lacson & Jones, 2016; Van Buskirk et al., 2017). More effort is needed to theorize on the processes through which OBMs operate despite the absence of favorable institutional conditions.

### Generativity in OBMs Infrastructures

Digital infrastructure, also called information infrastructure or cyberinfrastructure, is a term which encompasses a socio-technical interconnected structure of systems, people and organizations (Henfridsson & Bygstad, 2013). The extant literature on digital infrastructures has researched the phenomenon in several contexts, such as the development of the Internet (Hanseth & Lyytinen, 2010), mobile platforms (Eaton, Elaluf-Calderwood, Sørensen, & Yoo, 2015), websites and services for electronic commerce (Hanseth & Monteiro, 1997).

Digital infrastructures may be regarded as an organizational phenomenon; they include not only the technical solutions, but also the organizations and people who leverage the services (Braa, Hanseth, Heywood, Mohammed, & Shaw, 2007; Henfridsson & Bygstad, 2013; Tilson, Lyytinen, & Sorensen, 2010; Vaast & Walsham, 2009). An infrastructure is the result of a process by which multiple human actors translate and inscribe their interests and needs into a technology, creating an evolving network of human and nonhuman elements such as technologies, processes, standards (Aanestad & Jensen, 2011; Constantinides & Barrett, 2014; Hanseth & Monteiro, 1997; Yoo, Lyytinen, & Yang, 2005). Digital infrastructure and their architecture fuels platforms, websites and other artifacts, that exists within them thanks to the layered and modular nature of such complex systems (Constantinides et al., 2018; Yoo, Henfridsson, & Lyytinen, 2010).

The literature has highlighted some key attributes: (i) digital infrastructures are different from traditional information systems; they are heterogeneous, often with no dominant actor (Hanseth and Lyytinen 2010); (ii) the dynamics of digital infrastructures are also different in that they are not designed, since it has been proven that they evolve through innovation, adoption and scaling (Henfridsson & Bygstad, 2013); (iii) innovation is characterized by nonlinear evolutionary dynamics, it is hard to predict, and it is the result of the interrelations among a variety of actors (Bygstad, 2010).

Digital infrastructures evolve over time as a result of the generative processes that shape the evolution of such complex socio-technical artifacts (Henfridsson & Bygstad, 2013). Generativity is defined as the capacity to make difficult jobs easier, to offer additional kinds of uses, to easily use and access the technology (Zittrain, 2006). The notion of generativity has been introduced to explain innovation, rapid scaling and adaptation in digital infrastructures (Henfridsson & Bygstad, 2013; Huang, Henfridsson, Liu, & Newell, 2017). Generativity does not take place only in collaborative and favorable environments. As complex systems, digital infrastructures are exposed to major breakdowns determined by the propagation of local failures into large-scale disruptions (Hanseth & Ciborra, 2007). When digital infrastructures operate under adverse conditions, such as for instance in case of cyberattacks, generative processes are triggered to react to disruptions and breakdowns. Though longevity and durability are inherent properties of digital infrastructures (Tilson et al., 2010), the generative process and the underlying mechanisms through which they are achieved has been overlooked.

## Method

The OBMs phenomenon is characterized by a rich and complex set of elements. The technologies adopted are used for both illegal and law enforcement purposes: the same tools are used by communities of criminals, hackers and police agents making the boundaries of the phenomenon blurred. This makes the case study a suitable approach to investigate OBMs (Yin, 2018). We conducted a longitudinal case study: the longitudinal approach allowed us to conduct a process analysis (Berends & Deken, 2019) of how events unfolded over time and investigate the mechanisms explaining the infrastructure operations.

### Data Collection

The empirical context is represented by the digital infrastructures of OBMs, intended as the assemblage of technologies, actors and practices. We collected data from multiple sources; due to the characteristics of anonymity and secrecy of the analyzed markets and users, data triangulation and mixed method are more important than usual since one single source cannot give a reliable picture of the phenomenon (Ferguson, 2017). We adopted a mixed-method triangulation (Downward & Mearman, 2007), and we collected data referring to a period from 2012 to mid-2018. The aim behind the data collection was to obtain a full understanding of (i) events, (ii) actors, and (iii) technologies, as discussed below.

Regarding the events which occurred in the OBMs infrastructures, information has been obtained consulting reports from the police operations (e.g. EUROPOL), conducting open-ended interviews with police officers specialized in cybercrime and with representatives of governmental institutions such as the Council of Europe (details available upon request). Moreover, we also analyzed open sources of data on the Internet, i.e. historical data obtained accessing online forums, blogs, specialized webpages and public databases, that have been integrated with data reported in previous research papers.

Concerning the actors, we identified the following: hackers, site administrators, buyers, vendors and LEA’s agents. To investigate their characteristics, we collected data from hidden websites and police reports. Additional data on technological and behavioral trends have been collected by surveying a group of 32 experts working in forensics labs, criminal intelligence services, LEA’s cybercrime units and government Computer Security Incident Response Teams (CSIRT) from 20 separate EU countries attending a CEPOL course on cybercrime.

Finally, we investigated the technologies, i.e. the technological functions implemented to guarantee security and anonymity of transactions. Their diffusion and adoption have been traced and documented, integrating direct observation and historical data coming from secondary sources such as articles and posts published on websites, blogs, trade journals, newspapers, and online forums such as Reddit, often accessed thanks to the services of the Internet Archive (IA) website. We also collected technical information using scientific papers from the specialized literature (Horton-Eddison & Cristofaro, 2017). An overview of data collection strategy is shown in Table 2.

**Table 2:** Overview of data collection strategy

Topics	Code	Source of Data	Volume
Governmental and LEAs cooperation (Information about the context): - Details on LEAs intervention - LEAs priorities in contrasting cybercrime - Trends in financial frauds in Italy and Europe - Malware and vulnerabilities - Future trends in cybercrime - Attack scheme used by criminals	I1	Strategy briefs and reports from government, LEAs and professional associations	15 national cybersecurity strategies 6 ENISA threat landscape reports 5 EUROPOL IOCTA Reports 7 FBI IC3 reports 6 CLUSIT reports
	I2	Interviews with experts	4 LEA and military officers in charge of high-tech crime units 6 Chief Information Security Officers 4 Ethical hackers and security consultants 13 representatives of public institutions (e.g. Council of Europe, ENISA)
	I3	Scientific papers on criminology, drug trafficking, security and regulation	78 papers (49 in criminology journals, 22 in information systems journals, 7 others)
Technologies: - Establishment, close down and morphing of OBM - Details on communication, hacking and payments - On line discourse of community users - Actual trends in technological tools	T1	Longitudinal analysis of deepdotweb.com accessed through Internet Archive	277 webpages captured
	T2	Articles and posts published on websites, blogs; trade journals, newspapers	215 articles (52 Newspapers; 55 Trade journals; 69 Wire Feeds, 39 others)
	T3	Posts published reddit.com	1800+ posts
	T4	Darknet Market Archives	1,7 Terabyte of raw data
	T5	Questionnaire with LEA agents participating in a CEPOL training program on cybercrime	32 agents from 20 EU countries
OBM Offerings - Details of offerings in OBMs and forums - Technologies used for OBM offerings	C1	List of offerings related to credit cards and identity theft on sales in major OBMs	36 GB of data, up to18,831 pages/images per each OBM

### Data Analysis

We conducted a critical realist analysis, building on the method described by Bygstad, Munkvold and Volkoff (2016). The process is summarized in Table 3 and described as follows.

**Table 3:** In depth data analysis

Step	Output
1. Description of events	Timeline of disruptions as reported in Fig. 2
2. Identification of key entities	Actors: vendors, buyers, LEA agents, hackers, site admins. Objects: technologies for communication, hacking and payments, OBMs marketplaces. Full description in sections 5.1, 5.2 and 5.3.
3. Theoretical re-description (abduction)	Conceptualization of the Dark Net as a digital infrastructure (Hanseth and Lyytinen 2010)
4. Retroduction	Candidate mechanisms: (i) how structure influences action, (ii) how action triggers action, and (iii) how action reproduces or changes structure
5. Analysis of mechanisms	Selection of three mechanisms. See Figs. 5 and 6, full description in section 6.
6. Assessment of explanatory power	

**Fig. 1:**
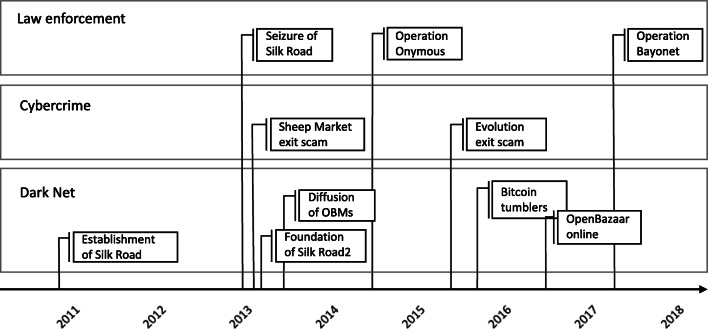
Timeline of events

Step 1 regards the description of disruptions that constitute the phenomenon of interest. Typical disruptions in the OBMs are, for instance, closure of a hidden site (e.g. Silk Road seized by FBI in 2012), diffusion of a new untraceable method of payment (e.g. the establishment of Bitcoin tumblers in 2011), emergence of a new business model for online commerce of illegal products (e.g. emergence of P2P markets such as Open Bazaar in 2016). Some events were well established in secondary sources, while other emerged from interviews with key informants. This procedure helped us establish a timeline of key events that occurred over time (see Fig. 1).

In the second step we identified the key entities (actors and objects) associated with the event (Volkoff, Strong, & Elmes, 2007). Key actors identified include hackers, vendors and buyers, site owners and admins and LEA agents. As objects, we refer to the technologies used in OBMs. Sections 5.1 and 5.2 report a description. In step 3, we analyzed the material to generalize in abstract terms the nature of the phenomenon and we conceptualized it using the digital infrastructure theoretical lenses, discussed in section 3.

The following steps 4–6 were conducted iteratively over a period of several months. Step 4 is retroduction and it consists of the exploration of various candidate mechanisms that could explain the observed outcomes. Following Hedström & Swedberg (1996), we looked for three types of mechanisms: how structure influences action (macro-to-micro), how action triggers action (micro-to-micro), and finally how action reproduces or changes structure (micro-to-macro).

The fifth step regards the analysis of mechanisms. In this phase we identified the candidate mechanisms and their relational entities, departing from the observed outcomes previously identified. Then, we worked to develop an explanation of the causal process based on interaction and dependency among interrelated entities and ensuing observable outcomes. Finally, we identified and documented three causal mechanisms that best explain the full breadth of observed events. The three identified mechanisms are: commoditization, platformization and resilience. A full description is offered in section 6.

The sixth and last step focuses on the assessment of explanatory power of the identified mechanisms. We treated the proposed mechanisms as a candidate explanation of digital infrastructure persistence, and we evaluated them against the empirical evidence. Finally, within our research team, we identified alternative mechanisms and we assessed them against our empirical evidences. These could explain some of the observed outcomes, but on their own were not satisfactory. The result of this analysis was that although several mechanisms were at work, only the three mechanisms described in section 6 were consistent with all the data.

## Empirical Evidences

The OBMs digital infrastructure is a socio-technical system consisting of technologies, organizations and actors. What distinguishes it from other infrastructures is the continuous challenges posed to his existence given by the presence of adverse conditions such as absence of formal rules, law enforcement control, legal protection, social legitimacy and conflicting goals among actors. This translates in the development of appropriate means to ensure users’ anonymity and in a highly dynamic and continuously changing nature. A number of significant events express such dynamism: growth of marketplaces, law enforcement interventions, changes in technologies for exchanging goods. In the remainder of the section, we present an overview of the technologies that enabled OBMs establishment as well as a review of the functioning of OBMs and the more important events. While discussing the evidence, we will refer to the topics identified in Table 2 using the code reported in the third column (e.g. code I2 when referring to interviews with experts).

### Anonymity Tools and Enabling Technologies

Since its inception, the Internet has provided users with an infrastructure enabling different forms of interactions. Group communications, private messages and file sharing are fundamental functions provided by many tools with different levels of sophistication. Online communities whose members are concerned with anonymity have different options to interact without disclosing their real identity. In addition to the possibility of accessing the web with a Tor browser (i.e. a free and open-source software that enable anonymous communication and web browsing), the use of aliases and the establishment of private spaces for social interaction are common practices for OBMs users (source T3 in Table 2). Protocols and systems for distributed discussion system and online messaging created in the early 90’s’ such as the IRC and USENET (the bulletin board system) are still in use by hackers and criminals to exchange private messages (source T2 in Table 2). These tools are used in combination with web forums and blogs. An example is Reddit.com, a popular social news aggregator, established in 2005, which also hosted from 2010 until 2018 a subcommunity of approximately 20,000 members focused around OBMs related matters.

Since the Internet has no inherent cryptographic security, to ensure the secrecy of data exchange different solutions have been developed over time. These tools have been widely adopted in OBMs communications. For instance, Pretty Good Privacy (PGP) is a tool developed by political activists in the 90s and used today by Internet users including criminals for signing and encrypting all sort of data such as texts, email, files and directories (source I2 in Table 2). Encryption is also a key component of distributed ledger technologies (i.e. blockchain), the revolutionary solution for transaction processing. The most impactful blockchain application is the development of cryptocurrencies, such as the Bitcoin. After its launch in 2009, Bitcoin replaced previous payment systems used by criminals (Böhme, Christin, Edelman, & Moore, 2015). More recent developments of e-payment systems obfuscate the sender and the recipient of cryptocurrency transactions by tumbling wallets (i.e. shuffling a bundle of transactions together to disguise the origin of the funds. Examples of tumbling wallets are Dark Wallet, Bitcoin Fog) and include advanced cryptographic functions (e.g. Zcash) (source T2 in Table 2). The OBMs infrastructure is the result of a combination of the above-mentioned anonymity tools, developed and integrated to respond to emerging needs of the OBMs community.

### The Establishment and Evolution of OBMs Marketplaces

**Fig. 2:**
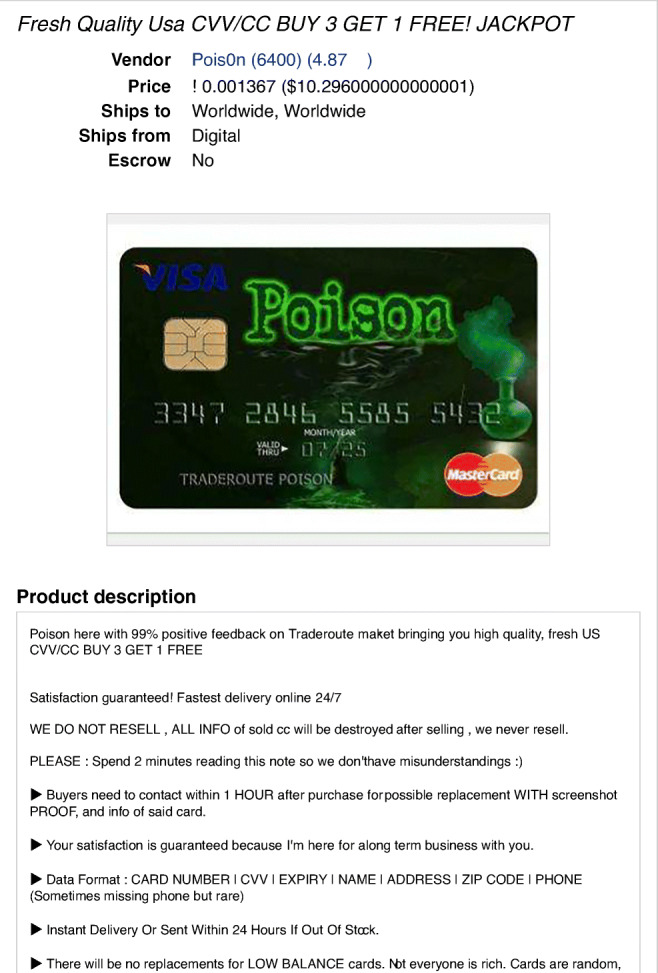
An example of offer taken from source C1 in Table 2

The first OBMs marketplace was SilkRoad, established in January 2011 and active for 33 months (source T2 in Table 2). SilkRoad was the first market established in the Tor network organized as an e-commerce platform. SilkRoad served mainstream clients with an anonymous, accessible method for purchasing illegal goods. Taking advantage of the anonymity guaranteed by the Tor infrastructure, it allowed the exchange of illegal goods and paved the road for further development of online crime (source T1 in Table 2). The success of Silk Road has shaped the structure of OBMs marketplaces: they are organized in two sided platforms and provide escrow functions similar to those available in conventional e-commerce websites. Payments are performed using cryptocurrencies and vendors use PGP keys for building their reputation while remaining anonymous. The typical offer includes picture to display the good, customer rankings of the vendor, payment system accepted, and escrow mechanism for secure exchange. We show in Fig. 2 a page from an OBM marketplace displaying the offering of a stolen credit card. As can be noted, the site mimics the logic of legitimate e-commerce sites, with product description, shipping information and payment systems.

The trade of illegal goods is conducted through anonymous transactions and shipping. To build trust between vendors and buyers, buyers are called to rate the vendors. In OBMs marketplace, building trust is central, as we can see from the buyer’s guidelines reported on Dream Market: *“First of all, all members are kindly asked to be honest regarding package, delivery, product quality and shipping conditions. This helps maintaining a trusted network, which is a major basis in hidden web marketplaces. Scammers are not tolerated and are quickly identified as such”* (http://xsuee6v24g2q6phb.onion/ help accessed on Dec 03, 2018 - source C1 in Table 2). Payment is usually performed through the use of escrow services; i.e. the marketplace admin withhold the payment until the buyer confirms the receipt of the goods. In our data, most of the shipping was done by regular mail (source C1 in Table 2).

**Fig. 3:**
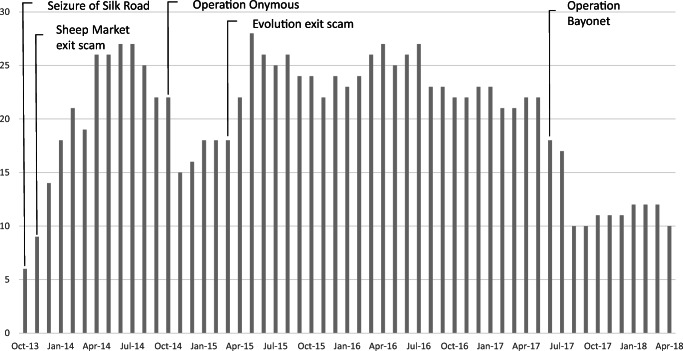
OBMs on Tor (2013–2018)

After the seizure of Silk Road, in the period from October 2013 until April 2018, we registered the existence of 122 marketplaces. During this period, 9 have been closed by LEAs and 42 have been closed by admins with an exit scam (sources T1 and T4 in Table 2). An exit scam occurs when an established business stops shipping orders while continuing to receive payments for new orders. Figure 3 reports the number of active marketplaces on Tor per months and 5 significant key events discussed below.

- *Seizure of Silk Road:* Silk Road was seized by the FBI in October 2013. The FBI arrested founder and administrator of the site and seized $32 million in BTC from accounts related to Silk Road. By the time Silk Road was closed, there were 6 active marketplaces, where many vendors were already active. Vendors did not stop their business: buyers were able to verify that they were dealing with the same vendors by using the same PGP or other encrypted signatures and this facilitated the migration of users among marketplaces. Reputation of vendors remained untouched and trust in the infrastructure was not lost.
- *Exit scam performed by SheepMarket:* in November 2013 the administrator of SheepMarket, a marketplace operating since February 2013, performed an exit scam stealing $6 million in BTC. Exit scams are common in OBMs. The SheepMarket exit scam, however, is relevant for our analysis because of the reaction of the OBMs community of vendors and buyers. In fact, collaborating and exchanging information through online forums and using investigation techniques (e.g. tracing BTC in a tumbling service), the OBMs community managed to discover the identity behind the pseudonym of the scammer and made it publicly available in the net. The pursuit of the administrator was the result of the collaboration of the whole community, that generated a creative and collective solution to increase trust and reliability of the infrastructure, as this post from Reddit.com shows: “*If you don’t want me to pursue this thief then I won’t. To whoever wants to continue my work here it is. If this helped anyone feel free to donate” (posted on* *Reddit.com/SheepMarket**on Dec 2013).* In March 2015, the SheepMarket administrator was arrested (sources T1 and T3 in Table 2).
- *Operation Onymous*: Operation Onymous is an international law enforcement operation conducted in October 2014 that shut down a number of websites, including 7 marketplaces. The operation required international collaboration among LEAs from 17 countries. Similar to previous cases, after the closure, users migrated to alternative platforms. In fact, despite scams and law enforcement efforts, OBMs continue to proliferate (sources I1, T1 and T3 in Table 2).
- *Exit scam performed by Evolution*: in March 2015, the Evolution marketplace was closed down by the admin that performed an exit scam: the admin took all the money contained in the escrow, an estimated amount of $12 million in BTC. Moreover, the Evolution scam created a major discontinuity since at that time it was the 2nd largest marketplace. After this exit scam, we observe a peak of new marketplaces. Many smaller markets emerged to take advantage of the turbulence generated by the event (sources T1 and T2 in Table 2).
- *Operation Bayonet:* it is an international LEA operation targeting two of the biggest marketplace active in June 2017: AlphaBay and Hansa. At the time of the seizure, AlphaBay was the largest marketplace, with over 369,000 listings and 400,000 users. After the closure of AlphaBay, Hansa was projected to become one of the leading markets. However, on July 20th 2017 it was revealed that Hansa had been compromised by LEAs for several weeks before closing, shortly after AlphaBay’s seizure. Dutch police impersonated the site’s administrators, collecting usernames, passwords and addresses of Hansa buyers. After AlphaBay’s closure, the police allowed the Hansa user base (growing from 1000 to 8000 vendors per day, due to the AlphaBay shutdown) to make illegal transactions in order to collect evidence for future prosecution of users, as the Dutch Police declared on the list of FAQ they published on the Hansa webpage after seizure: “*Question: Why have you done this? Answer: Hansa Market was a darknet market that was primarily used to sell illicit goods. We have chosen to take over this site to collect as much information as possible on its users. Furthermore, we want to send a clear message that using darknet markets is not an anonymous activity.”* (retrieved from http://politiepcvh42eav.onion/hansafaq.html accessed September 07, 2017). After Operation Bayonet, we observe a reduction of active marketplaces to 10 (sources I1, I3 and T1 in Table 2).

After this event, there was a year of relative stability in the number of active marketplaces. Interviews with LEA operators and analysis of secondary data suggest that platform-like marketplaces are facing maturity and a new form of illegal marketplaces, the P2P commerce, was proliferating (source I2 in Table 2). P2P commerce, also called decentralized market, is an alternative form of trade illegal goods. Although not entirely new, decentralized markets are assuming new relevance since the launch in April 2016 of OpenBazaar (Chainanalysis, 2020). OpenBazaar is an e-commerce website that hosts a fully decentralized marketplace eliminating administrators in the markets. It is claimed that similar marketplaces enhance anonymity and safety: the lack of a central point of control that can be taken down, assures continuity in the services and eliminates the risk of exit scams or robbery. Moreover, no data about transactions and users are collected and aggregated and there is no censoring or control. We expect a continuous evolution in OBMs digital infrastructures: as one of the LEA agents interviewed said: “*whatever we invent, there will always be crime*” (source I2 in Table 2).

## Mechanisms

**Fig. 4:**
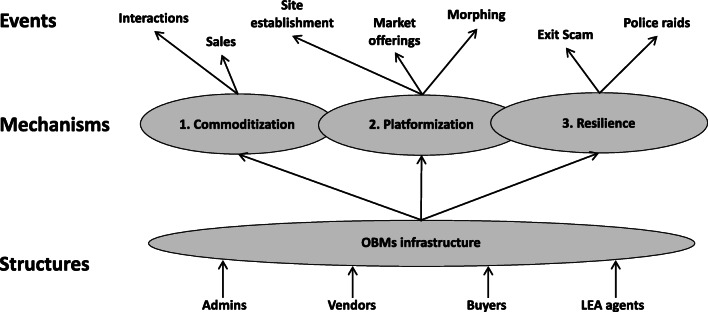
Events, mechanisms and structure

Through systematic retroduction we identified three high-level mechanisms of OBMs infrastructure. These mechanisms operate on the structural elements and leads to observable events. A schematic illustration is offered in Fig. 4.

### Mechanism 1: Commoditization

**Fig. 5:**
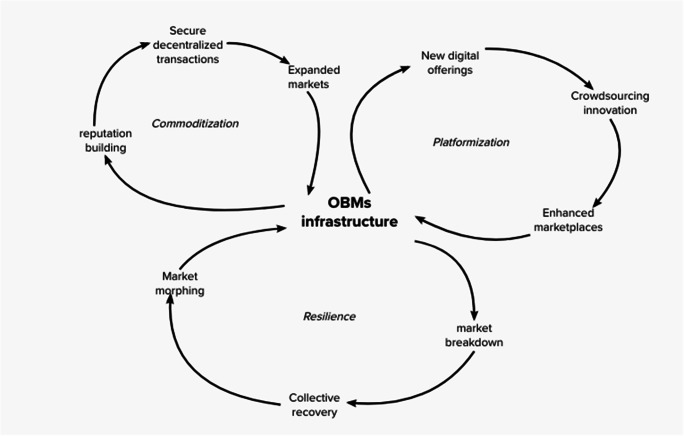
OBMs mechanisms

The first mechanism identified is “commoditization” (see fig. 5). The identification of this mechanism departed from the observation of OBMs’ normal functioning. The structure of marketplaces in OBMs is much the same as conventional OMs, enabling vendors and buyers to meet and trade at low transaction costs. Similarly to traditional OMs like Amazon and eBay, buyers can be victims of different forms of deception, such as for instance the non-delivery of items, product inauthenticity and misrepresentation. However, this risk is accentuated in OBMs, given the lack of transparency, law enforcement control, legal protection. It is not possible to appeal to trusted third parties in case of disputes, as it generally occurs when transacting in legal markets.

Despite these risks, evidence shows that online commerce of illegal goods is flourishing and successfully supporting buyers and vendors. How can the buyer, the vendor and the marketplace admin trust each other? Our data show that it is relatively easy for a buyer to browse offerings, select an object, purchase it anonymously and rate the vendor (source T1 in Table 2). Vendors can build their reputation by providing additional services and information to guarantee the quality of the purchase; as examples we can mention functions for checking the validity of stolen credit cards and refunding policies issued by vendors. Moreover, admins, in order to prevent deception, can implement advanced trust functions based on services and tools widely diffused in OBMs digital infrastructures (i.e. escrow). Given the absence of central actor trusted by all parties, the security of transactions is assured through decentralized systems such as cryptocurrencies and public key infrastructures. These arrangements imply a wider diffusion of illegal services. We use the term “commodity” to emphasize the standardized nature of services such as credit cards, botnet rental, phishing, etc. The increase in the diffusion of “commoditized” illegal services, attracts new buyers and vendors. The OBMs infrastructure benefits from additional buyers and vendors, since the quantity and the variety of goods increase. This process, in turn, attracts additional actors to create new offerings and generate an expanded marketplace in the OBMs infrastructure. We suggest calling this self-reinforcing mechanism “*commoditization*” (See Fig. 5, left hand side). We define *commoditization* as *a process where the OBMs infrastructure enables vendors to build their reputation and to assure the security of transactions through decentralized controls; successful purchases attract a critical mass of users to trade a greater variety of products and services.*

#### Mechanism 2: Platformization

The second mechanism identified is called *platformization* (See fig. 5)*.* We already discussed the role of the OBMs infrastructure as a powerful resource for enabling the commerce of illegal goods. In OBMs marketplaces, hackers develop and sell new versions of malware and exchange information with peers through secure communication channels. The multiplicity of digital goods and services available in OBMs marketplaces triggers new forms of illegal activities. For instance, datasets with personal data are sold to conduct personalized phishing campaigns and perpetrate fraud on a large scale through the use of cryptocurrencies to collect payments and for money laundering. Furthermore, marketplaces admins constantly monitor the fast-evolving landscape of digital solutions and adapt their marketplaces by integrating functions to respond to users’ needs. Thus, marketplaces are enriched with new functions to fulfill the emerging needs of criminal communities (i.e. crowdsourcing innovation). For instance, a core function of the OBMs infrastructure is represented by electronic payment systems that are based either on Bitcoin or other cryptocurrencies. This enables the marketplace in the OBMs infrastructure to enhance their offering. We use the term “platformization” to emphasize this evolution over time.

We define “*platformization*” as *a process where the OBMs infrastructure enables hackers to sell new digital goods and admins to enhance security and efficiency of transactions by implementing complementary features crowdsourced from external communities*.

The two mechanisms described above are the basic forces of OBMs infrastructure, making operations possible despite the lack of transparency, law enforcement control, legal protection and centralized governance. The two mechanisms interact closely; network effects enacted by the *commoditization* mechanism increase the quantity and variety of offering and generate the means through which the *platformization* mechanism is established and operates. *Platformization* allows the development of enhanced network resources to better satisfy service and content needs for the multiple users involved. However, the OBMs infrastructure is also characterized by disruptions. The main sources of these breakdowns are the occasional failure determined by admins, hackers and LEAs. These dynamics are the basis for the third mechanism called *resilience*.

#### Mechanism 3: Resilience

Specific characteristics of the OBMs infrastructure (i.e. anonymity, untraceability, lack of legal protection and illegality of goods exchanged) can lead to sudden and frequent interruptions of normal functioning. Such interruptions can be caused by unpredictable events such as an exit scam or a police operation. In the first case, the deceivers exploit the opportunities created by the presence in the escrow system of substantial amounts of money: the deceiver may transfer cryptocurrencies to his own account, and close down the site without a trace for neither vendors nor buyers. In the case of police operations, we observe the actions of LEAs that seize websites and marketplace and block the trade of illegal goods.

There are observable consequences of those events. For instance, the number of OBMs significantly declines after documented police operations. Other examples of consequences are the reaction of the communities of users triggered by Sheep Market and Evolution exit scams. In the former case, a collective action was conducted to discover and disclose the identity of the deceiver. Following the latter, an increase in number of active sites was observed. After a period of “collective recovering” during which different actions take place, we observe changes in both processes and technologies. For instance, after an exit scam, vendors move to more trustworthy OBMs with enhanced security functionalities. Similarly, criminals react to the strategies implemented by LEAs by experimenting new attack schemes based on the adoption of advanced tools such as peer-to-peer markets (e.g. OpenBazaar), more reliable payment systems and encrypted point to point channels for communication (e.g. PGP keys). Therefore, a resilient OBMs infrastructure is generated by the interactions between anonymous actors and enhanced digital tools technologies.

We define *“resilience”* as *the collective recovery actions undertaken by the user community resulting in the morphing of technologies and deception schemes in response to successful and unpredictable actions of actors with contrasting goals*.

The 3 mechanisms and their interactions are reported in Fig. 5.

## Discussion

Our empirical domain offers the opportunity to study the functioning of OMs under unique institutional conditions: frequent disruptions and presence of unknown actors with conflicting goals. We also observe the coexistence of heterogeneous and adverse forces influencing infrastructural growth: the interplay of technological and social structures in OBMs produces outcomes that are different from those of traditional digital infrastructures (e.g. Henfridsson & Bygstad, 2013) in which sources of disruption are competitors and new entrants. In OBMs infrastructures, opposing forces offset the self-reinforcing dynamics of *innovation*, *adoption* and *scaling*. *Innovation* is hampered by the reduced possibility to combine services that are hidden. *Adoption* is discouraged by deceptive behaviors of buyers and vendors. *Scaling* is prevented by law enforcement operations that limit the reach of the infrastructure. Notwithstanding these adverse conditions, the OBMs infrastructure continue to operate. Hence, we return here to our research question, *what are the generative mechanisms of the Online Black-Markets infrastructure?*

We identified three mechanisms – *commoditization*, *platformization* and *resilience* – that explain OBMs operations as the result of interactions between structural elements and individual actions. The first two mechanisms (i.e. *commoditization*, *platformization*) take place during regular market operations; they reveal in detail how the structuring of the illegal commercial transactions is key for the functioning of OBMs and show that the technical and social elements not only interact to facilitate transactions, rather they *constitute* OBMs, feeding on each other. The third mechanism (i.e. *resilience*) instead focuses on the reaction to unpredictable and abrupt changes in the infrastructure (such as admins exit scams or LEA operations); it also shows how resilience emerges from the collective action of users recovering after an infrastructure breakdown.

Together the three mechanisms describe the generative forces of the OBMs infrastructure. They simultaneously interact to enable infrastructure operations. Each individual mechanism fails in producing this outcome independently. For instance, commoditized illegal goods and services fail to reach buyers without a platform that guarantee secure transactions. Moreover, secure transactions require innovative solutions to solve the problem of decentralized control (Li & Whinston, 2020). Similarly, when marketplaces growth in size, they may attract the attention of LEAs that will eventually interrupt their operations. The OBMs infrastructure has to cope with similar shocks. Without a resilience mechanism to recover from disruptions, activities could not be carried out anymore, services would not be accessible any longer and marketplaces would fail in attracting new offerings. The co-existence of the three mechanisms is necessary to generate the OBMs infrastructure.

A fundamental aspect that characterizes the three mechanisms is that they all share a community orientation. Online communities collectively contribute to build the reputation of buyers and vendors. Also, the technical solutions that are plugged in the marketplaces are produced by communities of hackers and software developers (Flowers, 2008). Finally, the recovery process that morph a marketplace following a disruption is also performed online by anonymous members of online communities interacting via social media (Huang & Benyoucef, 2013; Spagnoletti et al., 2015). Members of these communities are temporarily engaged in problem-solving activities without necessarily expressing solidarity with other users. Community support and other forms of participation characterizing social commerce in OMs are also present in OBMs despite the limited trust among e-commerce users. Our findings show that networks and bottom-up information flows trigger actions for infrastructure formation while in traditional mechanisms individuals recombine, adopt or add solutions (Henfridsson & Bygstad, 2013).

The proposed model of OBMs mechanisms increases our understanding of e-commerce and social commerce phenomena. Rather than focusing on the identification of contingency factors, it explains how institutional and social factors interact and influence the performance of OMs. Our model adds a process view to explain the role of social media in e-commerce (Huang & Benyoucef, 2013). It also challenges the current understanding on the role of institution-based trust in social commerce (Lu et al., 2016). In fact, it shows that proper transactional conditions in e-commerce can be created without the involvement of authorities and trusted third parties. Future studies may investigate these issues further to see how trust emerges as a combination of transaction, innovation and resilience behaviors, leading to a new definition of trust in social commerce (McKnight et al., 2002). The role of institutional structures can also be empirically explored in online contexts characterized by adverse conditions (Gefen & Pavlou, 2012). Furthermore, from the perspective of the marketplace administrator, future studies can address the problem of platform governance and control to balance transaction, innovation and resilience in platform ecosystems (Ceci, Prencipe, & Spagnoletti, 2018; Constantinides et al., 2018; de Reuver et al., 2017).

Our view of OBMs as digital infrastructures, complements the current understanding on value-creation in OBMs. Our mechanisms show that transaction and innovation dynamics are intertwined in OBMs marketplaces. While value chain and transaction cost models shed light on the structure of criminal networks (Bakken, Moeller, and Sandberg 2017; Huang et al. 2018; Kraemer-Mbula, Tang, and Rush 2013; van Wegberg et al. 2018), our model captures the dynamics of transaction and innovation processes that together generate the OBMs infrastructure. The OBMs operations rely on the successful execution of transactions whose security is assured by external computing and network resources, integrated in the infrastructure. Furthermore, additional external resources are mobilized to respond to failures and disruptions. OBMs’ resilience relies on external resources such as social media platforms (e.g. Reddit) that become temporarily part of the infrastructure to support a collective recovery process. Future studies can further investigate these processes by conducting content and network analysis of conversations on social media to reveal how cybercriminals coordinate for resilience (Décary-Hétu and Giommoni 2017; Lacson and Jones 2016).

The resilience mechanism of OBMs, shows that users engage in the framing of collective actions and beliefs that legitimize the activities of a collective, in absence of a central structure establishing uniform rules for all settings. Further investigations on the framing strategies and the pragmatic, cognitive and moral legitimacy in place, can offer unique insights on how a polycentric approach to governance may support successful infrastructure development and scalability. Therefore, future studies can focus on the development of digital infrastructures where data control is the outcome of collective action processes involving heterogeneous interests and resources of a distributed user base (Constantinides & Barrett, 2014).

﻿ Our study also has important practical implications to face the societal challenges of civil security. We empirically support conjectures on the dynamics of innovation in illegal and online contexts and we advance previous studies by analyzing the phenomenon from an IS perspective. By looking at OBMs as a digital infrastructure we integrate the social and architectural aspects into a single view. We include criminal and law enforcement activities in a holistic model that shows the interdependency among different areas of illegal activities and their supporting technologies. We also conceptualize OBMs as crowd-powered catalyst organizations and recommend LEAs to monitor OBMs dynamics balancing the technological and the social dimensions. OBMs serves as digital innovation hubs for criminal communities and can be used to discover new schemas of illegal activities.

Finally, by focusing on OBMs resilience, the proposed model can be used as a reference tool for developing realistic scenarios on the effects of crowd-based infrastructure breakdowns. The outcome can be used to train LEAs and cyber-intelligence units. Our longitudinal and critical realist approach sheds lights on the processes that generate OBMs. The knowledge on the generative mechanisms can inspire the search for innovative lawful societal and technological means for preventing, detecting and investigating new forms of crimes.

## Conclusion

In this paper, we investigated the OBM infrastructure through an in-depth analysis of its generative mechanisms. We offer two main contributions. Firstly, we advance the understanding on OMs by analyzing OBMs, a unique digital infrastructure characterized by the absence of formal rules, legal protection and social legitimacy and the resulting negative global impact of its social outcomes. The OBMs’ infrastructure is fueled by sociotechnical interactions and frequent shocks and operates despite the efforts of law enforcement institutions to suppress or eliminate it. Our study enabled us to identify three mechanisms that explain how OMs can operate under conditions of frequent and unexpected change. Secondly, by focusing on the community-based nature of the three mechanisms, we conceptualize OBMs as crowd-powered catalyst organizations and we discuss the implications for research and practice. We also offer methodological guidance to scholars and practitioners interested in making sense of observable events in this complex domain. Our three mechanisms explain OBMs’ operations and provide actionable knowledge for e-commerce and law enforcement. Notwithstanding the continuous evolution of OBMs and the limited timeframe of our data collection, our theoretical model also applies to OBMs’ phenomena emerging in the last three years. Thus, the evidence collected in seven years of OBMs’ evolution provided sufficient input and support to both complete the retroduction phase of our analysis and positively assess the explanatory power of the three mechanisms. Future studies can replicate our findings by applying the model to new impactful events such as recent marketplace seizures and Covid-19 related offerings.
